# A Reference Standard for Analytical Testing of Erythropoietin

**DOI:** 10.1007/s11095-022-03213-1

**Published:** 2022-03-15

**Authors:** Huiping Tu, Kevin Carrick, Rebecca Potts, Mark Hasselberg, Mark Verdecia, Chris Burns, Ben Cowper, Fouad Atouf

**Affiliations:** 1 United States Pharmacopeial Convention, 12601 Twinbrook Pkwy, Rockville, Maryland 20852 USA; 2 National Institute for Biological Standards and Control, Blanche Lane, South Mimms, Hertfordshire, EN6 3QG UK

**Keywords:** Biologics, erythropoietin, lyophilization, manufacturing, reference standards

## Abstract

**Purpose:**

Erythropoietin (EPO) is a 165 amino acid protein that promotes the proliferation of erythrocytic progenitors. A decrease in endogenous EPO production causes anemia that can be treated with recombinant Human EPO (rHuEPO).

**Objective:**

To ensure the safety and efficacy of the rHuEPO, manufacturers must use analytical methods to demonstrate similarity across batches and between different products. To do this they need reference standards to validate their equipment and methods.

**Method:**

We used peptide mapping, size-exclusion chromatography, glycoprofiling, and isoelectric focusing to analyze a rHuEPO reference standard.

**Results:**

Characterization demonstrates that our rHuEPO reference standard meets the criteria for quality.

**Conclusion:**

The rHuEPO reference standard is fit for purpose as a tool for validating system suitability and methods.

**Supplementary Information:**

The online version contains supplementary material available at 10.1007/s11095-022-03213-1.

## Introduction

Manufacturers of erythropoietin (EPO) biosimilars must demonstrate that their product is highly similar to and has no clinically meaningful differences from that of an already licensed reference product (RP). (1) This requirement assumes that the biosimilar manufacturer has access to batches of RP, as well as an in-depth understanding of the relevant critical quality attributes (CQAs). However, unlike the original developer of recombinant human erythropoietin (rHuEPO), the biosimilar manufacturer does not have extensive knowledge of the existing manufacturing process because it is often proprietary. (2) Some information can be gleaned from reports published by regulatory agencies, but acquiring direct knowledge of established controls and acceptance parameters is unlikely. (3, 4) The biosimilar manufacturer will therefore need to develop its own manufacturing processes (e.g., different cell lines, raw materials, equipment, procedures, process controls, and acceptance criteria). Under these circumstances, it can significantly benefit the biosimilar manufacturer to have access to a public reference standard (RS) for EPO that can be used to validate methods and qualify systems. An RS for EPO, combined with some knowledge of the process and expertise in analytical characterization, can form the basis of a biosimilar manufacturing strategy (Table I).

**Table I:** Biosimilar Reference Product vs. Biological Reference Standard

	Biosimilar Reference Product (RP)	Biological Reference Standard (RS)
**Role**	Define quality attributes for: 1. Similarity 2. Clinical characteristics 3. Comparability	Measurement tool across: 1. Different laboratories 2. Materials & Methods 3. Time
**Presentation**	Dosage form with defined shelf life representative of a single manufacturer	Formulated for long-term fitness for use with products from several manufacturers
**Potential Drift**	Products can drift and evolve due to manufacturers’ changes	Reference standards are designed to be resistant to drift
**Availability**	It depends on the innovator or supplier of original material	Assured continuously by the responsible organization

Biosimilar developers also need to perform comparability studies to assess the quality attributes of their biosimilar candidate against the RP. An RS cannot substitute for an RP for this purpose, but it can be useful for establishing the assays necessary for evaluating the similarity of the candidate biosimilar. These assays are highly scrutinized by regulators because they serve as the basis for any claims of biosimilarity. In the United States, comprehensive comparative analytical data are necessary to demonstrate biosimilarity and interchangeability under section 351(k) of the Public Health Service Act, and a “*Marketing Authorisation Application”* is required under Article 10(4) of Directive 2001/83/EC in Europe. (5, 6) The results of the comparability exercise need to be of such quality as to allow the regulatory agency to draw definitive conclusions about the similarity of the physicochemical, biological, and pharmacological attributes between the therapeutic product under review and the originator product, but also across batches when reviewing post-approval changes. Only then can the manufacturer claim that their product is highly similar and has no clinically meaningful differences. (7)

Manufacturers of EPO need to validate the physicochemical properties and the activity of their product. The identity should be validated by peptide mapping and the presence of high-molecular weight species should be monitored using size-exclusion chromatography. The glycosylation of EPO should be checked by HILIC HPLC and isoforms by isoelectric focusing. To assist developers with these tasks, we describe the development and characterization of USP’s recombinant human erythropoietin reference standard (USP rHuEPO-RS) for use in analytical comparability or system suitability tests.

## Materials & Methods

Bulk recombinant human erythropoietin drug substance (rHuEPO-DS) was produced at a commercial manufacturing site. Slide-A-Lyzer™ MINI Dialysis Units, 10 K molecular weight cut-off (MWCO), purchased from Thermo Fisher Scientific (Waltham, MA, USA), Amicon® Ultra-0.5 Centrifugal Filter Units, 10 K MWCO were purchased from Millipore Sigma (St. Louis, MO, USA), and 20 μg of Lysyl Endopeptidase, Mass Spectrometry Grade (Lys-C) was purchased from FUJIFILM Wako Pure Chemical Corporation (Richmond, VA, USA). PD MiniTrap G-10, Illustra NAP-10 columns, and Pharmalyte® 3–10 broad pH range ampholytes were purchased from G.E. Healthcare Life Sciences (Marlborough, MA, USA). Peptide-N-Glycosidase F (PNGase F) was purchased from New England Biolabs (Ipswich, MA, USA). The 2-AB Glycan Labeling Kit was purchased from QA-Bio, Inc. (Burlington, ON). QuikPrep® Macro SpinColumns™ G-10 was purchased from Harvard Apparatus (Holliston, MA, USA). Dithiothreitol (DTT), glacial acetic acid (p.a.), glycine, guanidine hydrochloride, high-resolution ampholytes pH 3.0–5.0, HPLC-grade water, L-Arginine, L-Histidine, methyl alcohol, Tween®20, phosphate-buffered saline (PBS) tablets, potassium persulfate, potassium chloride, potassium phosphate monobasic, potassium phosphate dibasic, sodium acetate, sodium citrate (Na_3_C_6_H_5_O_7_), sodium chloride, sodium hydroxide, sulfuric acid, 5 x concentrate fixing solution (60% (w/v) trichloroacetic acid and 17.5% 5-sulfosalicylic acid, trifluoroacetic acid (TFA), Tris and hydrochloric acid (HCl) were purchased from Millipore Sigma (St. Louis, MO, USA). Acrylamide (30%), bis-acrylamide (2%), and urea were purchased from National Diagnostics (Atlanta, GA, USA). Ammonium formate (3.8% Formic Acid) was purchased from Waters Corporation (Milford, MA, USA). Acetonitrile (ACN, ≥99.5% purity) and Acetic Acid were purchased from Thermo Fisher Scientific (Waltham, MA, USA). All chemicals for high-performance liquid chromatography (HPLC) were of analytical grade unless stated otherwise.

### Preparation of rHuEPO Reference Standard

Samples of rHuEPO-DS were reformulated, and 0.4 mL volumes were aliquoted into separate vials. The aliquots were then lyophilized to create the USP Erythropoietin Reference Standard (USP rHuEPO-RS). Each vial of USP rHuEPO-RS contains 100 μg of recombinant human erythropoietin, 12 mg Trehalose, 1.2 mg Arginine, 1.8 mg NaCl, 0.04 mg Tween®20, and 0.96 mg Sodium Phosphate dehydrate.

### Buffer Exchange

Buffer exchanges were performed by dialysis or spin-concentrator. For dialysis, the sample was transferred to the Slide-A-Lyzer™ unit, which was then floated in a beaker containing 100 mL of dialysis buffer for one hour at 5°C with low-speed stirring. The dialysis buffer used was based on the analysis to be performed after buffer exchange (see Results). After one hour, the dialysis buffer was discarded and replaced with cold fresh dialysis buffer. The dialysis was repeated five times, and the sample was subsequently removed from the Slide-A-Lyzer™ and transferred to a 1.5 mL microcentrifuge tube, and stored on ice. For spin concentration, 500 μL of dialysis buffer was added to an Amicon® Ultra-0.5 Centrifugal Filter Unit (Millipore Sigma) and centrifuged at 14,000×*g* for 15 min at room temperature. The sample was then added to the spin concentrator, followed by 450 μL of dialysis buffer, and then centrifuged at 14,000×*g* for 15 min at ambient temperature. The concentrator was washed with another 450 μL of dialysis buffer and then centrifuged again. The wash step was repeated twice more, with the flow-through discarded after each spin. The Amicon®Ultra filter device was separated from the microcentrifuge tube and placed upside down in a clean microcentrifuge tube. The sample was then centrifuged for 2 min at 1000×*g*. The concentrated sample was then transferred from the device to the tube and stored on ice.

### Peptide Mapping

rHuEPO-DS and USP rHuEPO-RS (100 μg) were digested with Lys-C following resuspension to 0.2 mg/mL in 1.0 M Tris (pH 7.3) at a protein to enzyme ratio of 20:1 (w/w) at 37°C for 30 min. Reactions were quenched with an equal volume of 8 M guanidine hydrochloride. The Lys-C–digested peptides were separated with a ZORBAX Eclipse XDB-C8, 3.0 × 250 mm, 5 μm, Agilent, USA) column in an Acquity ultra-performance liquid chromatography (UPLC) system (Waters, USA). Mobile phases A and B were 0.15% TFA in water and 0.12% TFA in 90% acetonitrile, respectively. A linear gradient used was 10–22% B for 30 min, 22–42% B for 80 min, 42%–65% B for 20 min, and 65–90% B for 0.1 min. The flow rate was 0.2 mL/min, and the column temperature was 30°C. Peak retention times were monitored using ultraviolet (UV) absorbance at 214 nm. rHuEPO digested with Lys-C is expected to produce 10 major peaks. The relative peak height percentage (RH%) of peak 8 and peak 9 from rHuEPO-DS and USP rHuEPO-RS were calculated as $RH{\%}_{peak\ 8\ or\ 9}=\frac{ peak\ height\ of\ peak\ 8\ or\ 9}{ peak\ height\ of\ peak\ 5}$ . The ratio of RH% (RRH%) for peaks 8 and 9 was calculated as $RRH\%=\frac{RH\% of\ rHuEPO- DS}{RH\% of\ rHuEpo- RS}$ .

### Size-Exclusion Chromatography-High Performance Liquid Chromatography

rHuEPO-DS and USP rHuEPO-RS were separated using a TSKgel® G3000 SW_XL_ (7.8 mm × 30 cm, 5 μm) column (Tosoh, Japan) connected to a Waters Alliance 2690 HPLC (Waters, USA). The column was equilibrated in running buffer (20 mM sodium citrate and 100 mM NaCl, pH 6.9), filtered using a 0.45 μm filter (Millipore Sigma, USA). rHuEPO samples were prepared at 1.6 mg/mL in running buffer and stored at 4°C in the autosampler before injection. 50 μL of samples were injected onto the column and separated in isocratic mode at a flow rate of 1 mL/min for 30 min at 25°C. Peak retention times were monitored using UV absorbance at 230 nm. Data acquisition and analysis were performed using Empower software (Waters, USA).

### Glycoprofiling

*N*-glycans were released from 100 μg of bulk rHuEPO-DS and USP rHuEPO-RS via incubation with PNGase F (1 U/mL) in 50 mM sodium phosphate (pH 7.3) and 50 mM DTT at 37°C for 30 min. Samples were then frozen in dry ice and dried by centrifugal evaporation using miVac (GeneVac™) with no heat. The released *N*-glycans were labeled with 2-AB according to the manufacturer’s protocol (QA-Bio, Inc., 2017). Briefly, 150 μL of glacial acetic acid (QA-Bio, Inc.) was added to a vial of 350 μL of dimethyl sulfoxide (DMSO) (QA-Bio, Inc.). The solution was mixed by pipette action. Then 100 μL of the DMSO-acetic acid solution was added to a vial containing 5 mg of LudgerTag™ 2-AB Dye (QA-Bio, Inc.). The solution was mixed until the complete dissolution of the dye. The total 100 μL volume of solubilized dye was added to a vial of LudgerTag™ Sodium Cyanoborohydride (QA-Bio, Inc.) and mixed by pipette action to create a labeling solution. The labeling solution was incubated at 70°C for up to 2 min and then cooled at room temperature for 10 min. Within 1 h of preparation, 5 μL of labeling solution was added to each EPO sample. The labeling reactions were mixed and incubated at 60°C for three hours. The excess 2-AB reagent was removed by cellulose disc solid-phase extraction. The *N*-glycans were eluted with water and thoroughly dried using a centrifugal evaporator. Briefly, 120 μL of water was added to each sample, followed by desalting using a G-10 gel filtration stationary phase resin prepared according to the manufacturer’s recommendation. Samples were centrifuged at 200 x *g* for 1 min. The flow-through was collected and passed through a second G-10 gel filtration by spinning at 200 x *g* for 1 min. The final flow-through (approximately 100 μL) was transferred to HPLC vials for analysis.

The 2-AB labeled *N*-glycans were separated by high-performance anion-exchange chromatography with pulsed amperometric detection (HPEAC-PAD) with a Dionex™ CarboPac™ PA100 4 × 250 mm column, a particle size of 10 μm (Thermo Scientific, USA) on a Dionex ICS-5000^+^ Dual Pump (Thermo Scientific, USA). Samples were analyzed with the Dionex™ ICS-5000^+^ ED Electrochemical Conventional Electrodes (Thermo Scientific, USA). The chromatography mobile phases (A, B, and C) were water, 0.5 M sodium acetate, and 0.5 M sodium hydroxide, respectively. The column temperature was 25°C, and the autosampler temperature was 4°C. The runtime was 130 min. The series of linear gradients were 10% B and 10% C for 15 min, 10–30% B and 10% C for 55 min, 30–90% B and 10% C for 24 min, 90% B and 10% C for 5 min, 90–10% B and 10–90% B for 6 min, 10% B and 90% C for 5 min, 10% B and 90–10% C for 1 min. The column was re-equilibrated in 10% B and 10% C for 29 min. The flow rate was 0.5 mL/min, and the fluorescence detection was set to 330 nm (excitation) and 420 nm (emission).

### Isoform Analysis

Isoelectric focusing was performed on a single horizontal gel. Briefly, a 6% T/0.16% C acrylamide-bis-acrylamide solution was prepared containing 5 M urea, 0.6% (w/v) 3–10 ampholyte, and 1.5% (w/v) 3–5 ampholyte. The acrylamide solution was passed through a 0.45 μM filter. Polymerization was initiated by adding potassium persulfate to a final concentration of 0.05% (1.8 mM). A 0.5-mm thick gel was cast between two glass plates (25.6 cm × 12.8 cm, 3 mm). Electrophoresis was run on the Multiphor II Electrophoresis System (G.E. Healthcare Life Sciences, USA) using 200 mM L-histidine as the cathode solution and 0.2 N sulfuric acid as the anode solution. The gel was pre-focused at 10 W for 20–40 min at 2–8°C. After pre-focusing, 15.0 μL of each sample was loaded onto the gel at the cathode site. The samples were focused at 10 W for 2.5 h at 2–8°C. The gel was incubated in a fixing solution (12% (w/v) trichloroacetic acid, 3.5% 5-sulfosalicylic acid, 40% methanol, and 10% glacial acetic acid) for 15 min. Fixing was repeated for an additional 15 min in the new fixing solution. The gel was then incubated in a wash solution (40% methanol and 10% glacial acetic acid) for 30 min and then stained for 60 min in 1.5 mM Coomassie Brilliant Blue R-250 dissolved in the wash. The gel was destained in 7.5% methanol and 10% glacial acetic acid until the sample bands were visible.

The gel was imaged, and densitometry was performed using ImageJ (National Institutes of Health). The profiles of the lanes were represented as the average of the grayscale values or the uncalibrated optical density along a one-pixel-height horizontal lane. A best-fit line was generated across the baseline of the profile. Peak boundaries were set as the troughs between adjacent peaks. Peak areas were then calculated for each individual peak. The peak areas within a lane were summed for the total area, and the percent contribution of each isoform was determined as the quotient of individual peak areas and the total area.

## Results & Discussion

### Peptide Mapping

The International Council of Harmonization (ICH) specifies peptide mapping as a critical quality test for confirming product identity, as a CQA for lot release. (8–10) Not surprisingly, it is also a necessary test for demonstrating the comparability of biosimilars to originator products. Therefore, peptide mapping was performed on the reformulated USP rHuEPO-RS to demonstrate its similarity to the source bulk drug substance (DS).

**Fig. 1:**
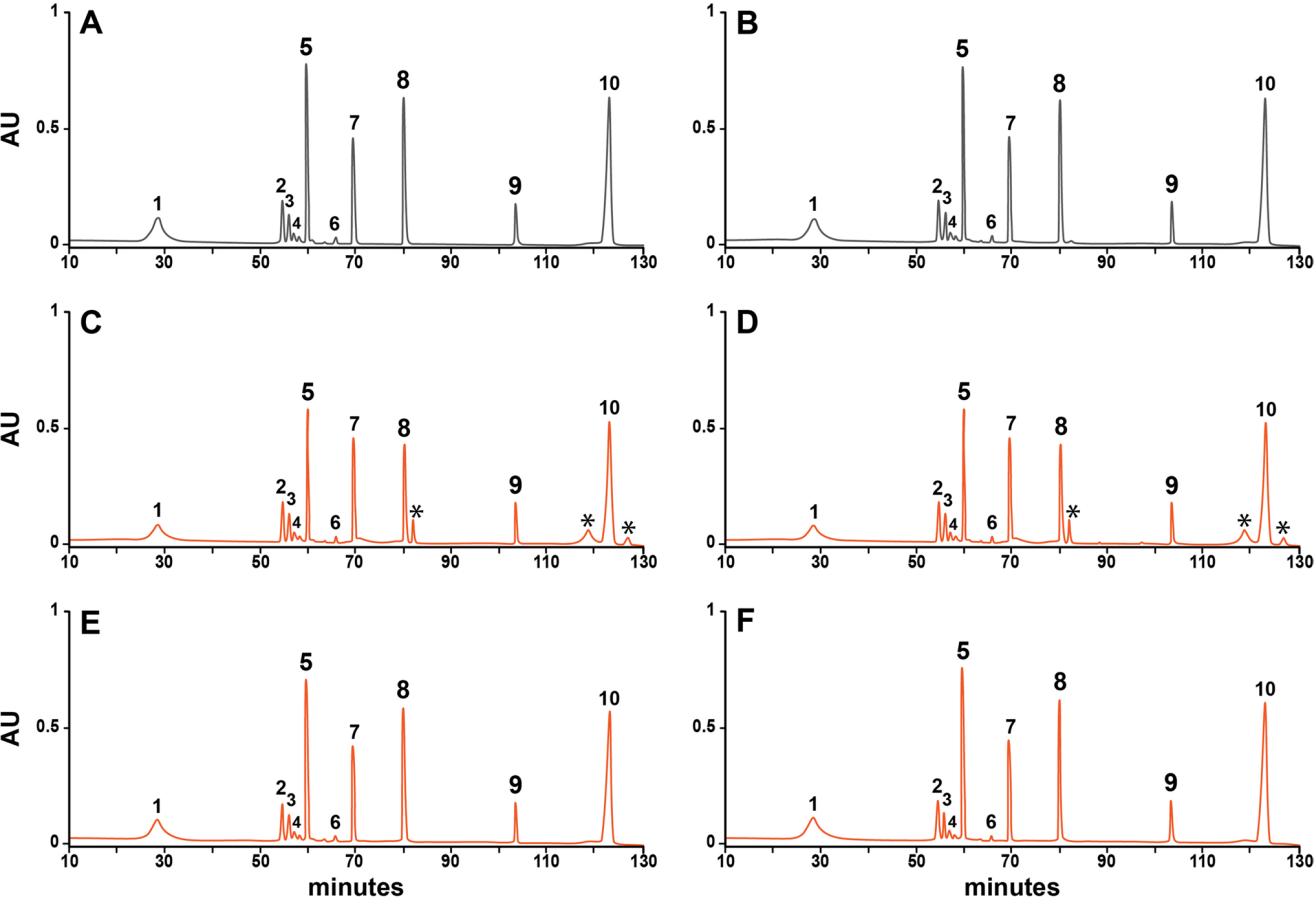
Peptide mapping. 100 μg of rHuEPO-DS (grey) and USP rHuEPO-RS (orange) were digested with the enzyme Lys-C and separated by UPLC. (A) rHuEPO-DS prepared with water, (B) rHuEPO-DS prepared with PBS; (C) USP rHuEPO-RS resuspended with water, (D) USP rHuEPO-RS resuspended with PBS, (E) USP rHuEPO-RS buffer exchanged with dialysis, (F) USP rHuEPO-RS buffer exchanged with a spin concentrator. Expected peaks are labeled 1 through 10. Unknown peaks are denoted with an asterisk (*).

Both rHuEPO-DS and USP rHuEPO-RS proteins contain eight lysine amino acids and two disulfide linkages that were not reduced prior to digestion with the protease Lys-C. The expected nine peptide fragments were separated using reverse-phase chromatography (Fig. 1). The chromatographic separation of the peptide fragments can be variable, so manufacturers rely on comparative testing between their sample and the reference material in side-by-side experiments. Therefore, only the chromatographic profiles between rHuEPO-DS and USP rHuEPO-RS were compared.

Enzymatic digestion of rHuEPO-DS produced a total of ten peaks (Figs. 1A & 1B). The first peak (peak 1) in each chromatogram is aggregate protein eluting near the column’s void volume (V_0_). The remaining nine peaks (peaks 2 through 10) represent the expected elution profile for the peptide fragments. However, the chromatographic profile for the newly formulated USP rHuEPO-RS displayed three additional peaks (Figs. 1C & 1D). The high concentration of excipients present after USP rHuEPO-RS is reconstituted in water may affect enzymatic digestion. To minimize the impact of excipients, an aliquot of lyophilized USP rHuEPO-RS was resuspended in water and then buffer exchanged into 1 M Tris (pH 7.3) by dialysis, or spin concentrator, before digestion with Lys-C and separation via UPLC.

The chromatogram of the buffer exchanged USP rHuEPO-RS produced the expected ten peaks. The retention times for each of peaks 5, 8, and 9 were within ±1.2 min of the corresponding peaks in rHuEPO-DS (Figs. 1E & 1F), thereby confirming comparability. The ratio of relative peak height percentage (RRH%) was within the acceptance criteria of 94–106%, and there were no additional peaks that were greater in height than peak 6. These results are consistent with the need to use buffer exchange, either by dialysis or spin concentrator, to achieve the expected peptide mapping profile for lyophilized USP rHuEPO-RS.

### Oligomerization

rHuEPO is predominantly monomeric when stored at 2–8°C. (11, 12) However, high-molecular-weight (HMW) species can form when the product is exposed to higher temperatures or certain stress conditions, and these product-related impurities negatively impact quality, safety and efficacy. (13, 14) Therefore, the stability of rHuEPO must be monitored using analytical methods that can resolve oligomeric forms from monomeric forms, such as size-exclusion high-performance liquid chromatography (SEC-HPLC).

**Fig. 2:**
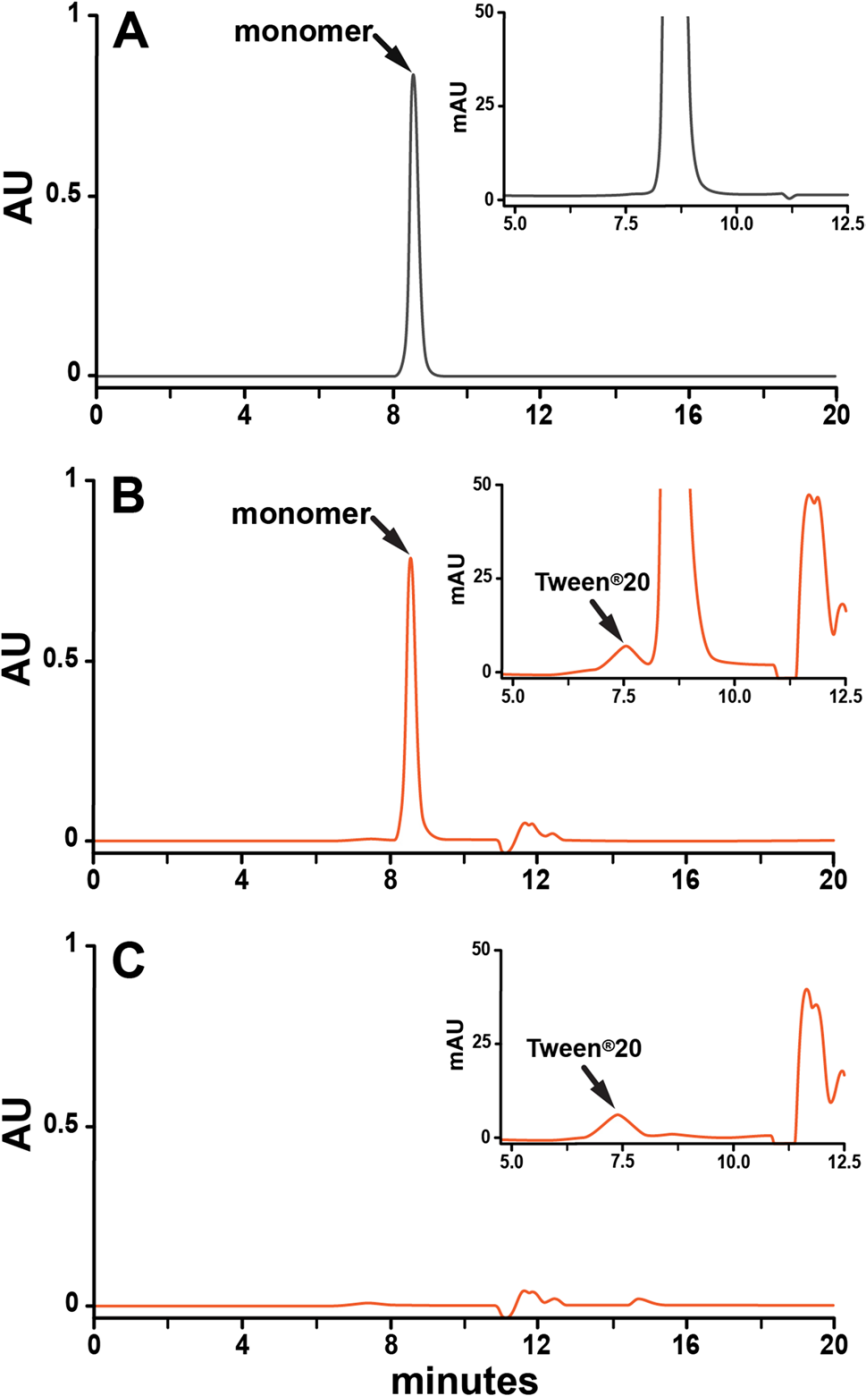
Analysis of rHuEPO monomer. 80 μg of rHuEPO-DS (grey) and USP rHuEPO-RS (orange) and an equivalent amount of excipient to that in 80 μg of USP rHuEPO-RS (orange) were analyzed by size-exclusion chromatography. (A) rHuEPO-DS, (B) USP rHuEPO-RS resuspended in water. (C) Equivalent amounts of excipients to (B) but without USP rHuEPO-RS. Monomer and Tween®20 peaks are labeled. *AU* is absorbance units at 230 nm.

**Fig. 3:**
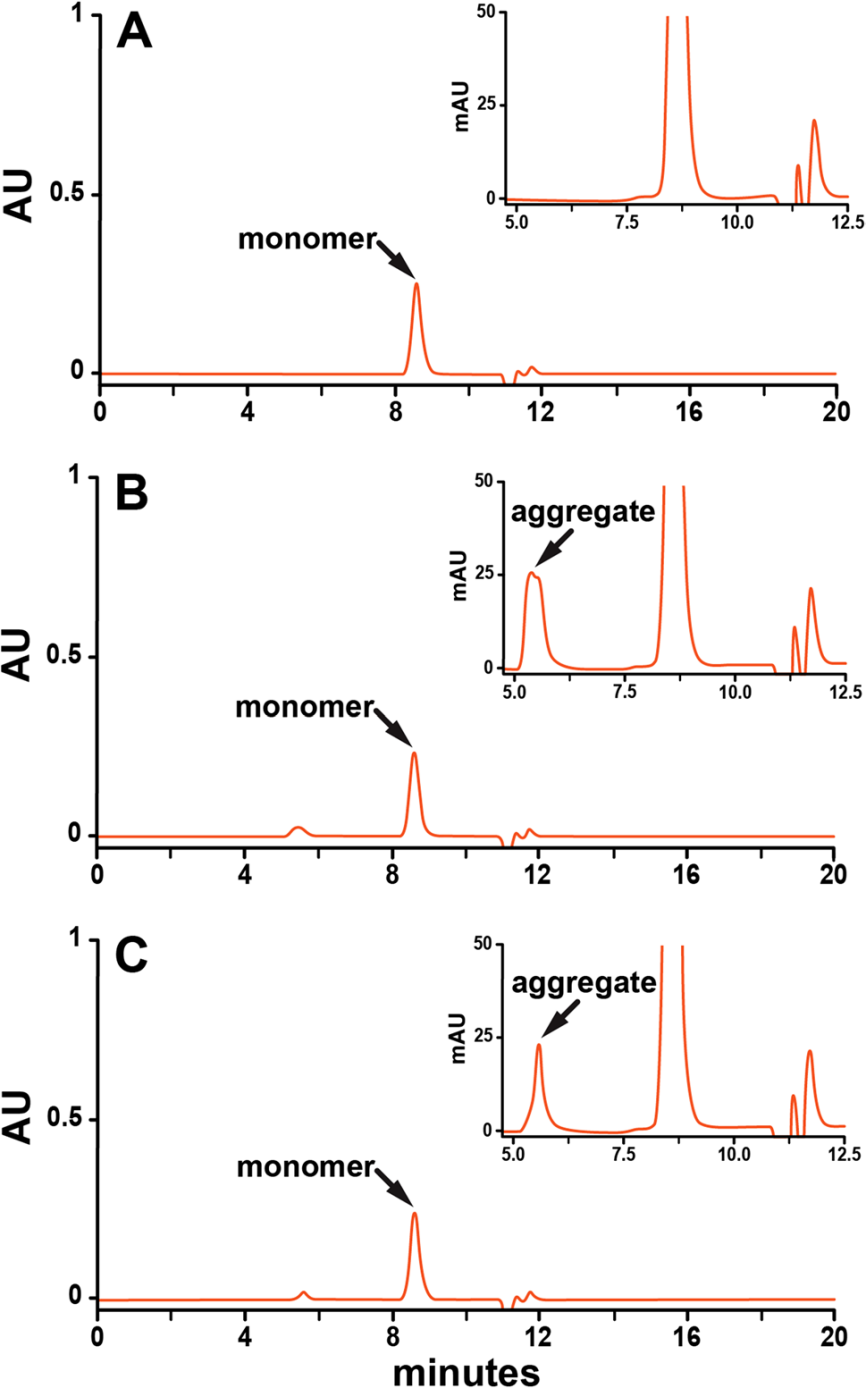
Analysis of aggregates after lyophilization. 25 μg of USP rHuEPO-RS (orange) was analyzed by size-exclusion chromatography. (A) no lyophilization, (B) lyophilization without Tween®20, (C) lyophilization with 0.001% Tween®20. Monomer and aggregate peaks are labeled. *AU* is absorbance units at 230 nm.

The rHuEPO-DS sample is completely monomeric, as determined by SEC-HPLC (Fig. 2A, Table II). Analysis of the USP rHuEPO-RS sample resulted in a peak corresponding to the monomeric protein and an additional peak, representing 1.3% of the total protein, eluting near the expected molecular weight of an oligomer of EPO (Fig. 2B, Table II). Previous studies of rHuEPO monomers have shown that the use of non-ionic detergents at high concentrations can result in additional peaks that do not consist of protein but which elute with similar retention times to rHuEPO dimers. (15, 16) To investigate whether this additional peak is Tween®20, vials containing only excipient were resuspended to the same concentrations as those present in USP rHuEPO-RS and then analyzed by SEC-HPLC (Fig. 2C). As expected, the excipient-only sample reproduced the additional peak. Furthermore, the additional detergent-only peak completely disappeared when USP rHuEPO-RS was formulated without Tween®20. However, the removal of Tween®20 prior to lyophilization resulted in the aggregation of 14% of the total protein as compared to non-lyophilized samples (Fig.3A & 3B, Table III). Lyophilization with Tween®20 but at reduced levels (0.001%) decreased the aggregation but did not completely remove it (Fig. 3C). Therefore, to preserve the integrity of the EPO protein and for the long-term storage, 0.01% of Tween-20 was added to the formulation buffer before the lyophilization process.

**Table II:** Oligomerization state of rHuEPO-DS and rHuEPO-RS

	Tween®20 Peak % (7.4 min)	Monomer Peak % (8.6 min)
rHuEPO-DS	0.00	100.00
USP rHuEPO-RS	1.33	98.67

Percentages of each peak were determined as the quotient of the individual peak area and the total area of both peaks. Peak retention times are in (). *mAU* is milli-absorbance units at 230 nm

**Table III:** Effect of Non-ionic Detergent on rHuEPO-RS stability

	Aggregate Peak % (5.6 min)	Monomer Peak % (8.6 min)
USP rHuEPO-RS, no lyophilization	0.00	100.00
USP rHuEPO-RS, lyophilization, no Tween®20	14.17	85.83
USP rHuEPO-RS, lyophilization, 0.001% Tween®20	8.23	91.77

Percentages of each peak were determined as the quotient of the individual peak area and the total area of both peaks. Peak retention times are in (). *mAU* is milli-absorbance units at 230 nm

### Glycan Profiling

**Fig. 4:**
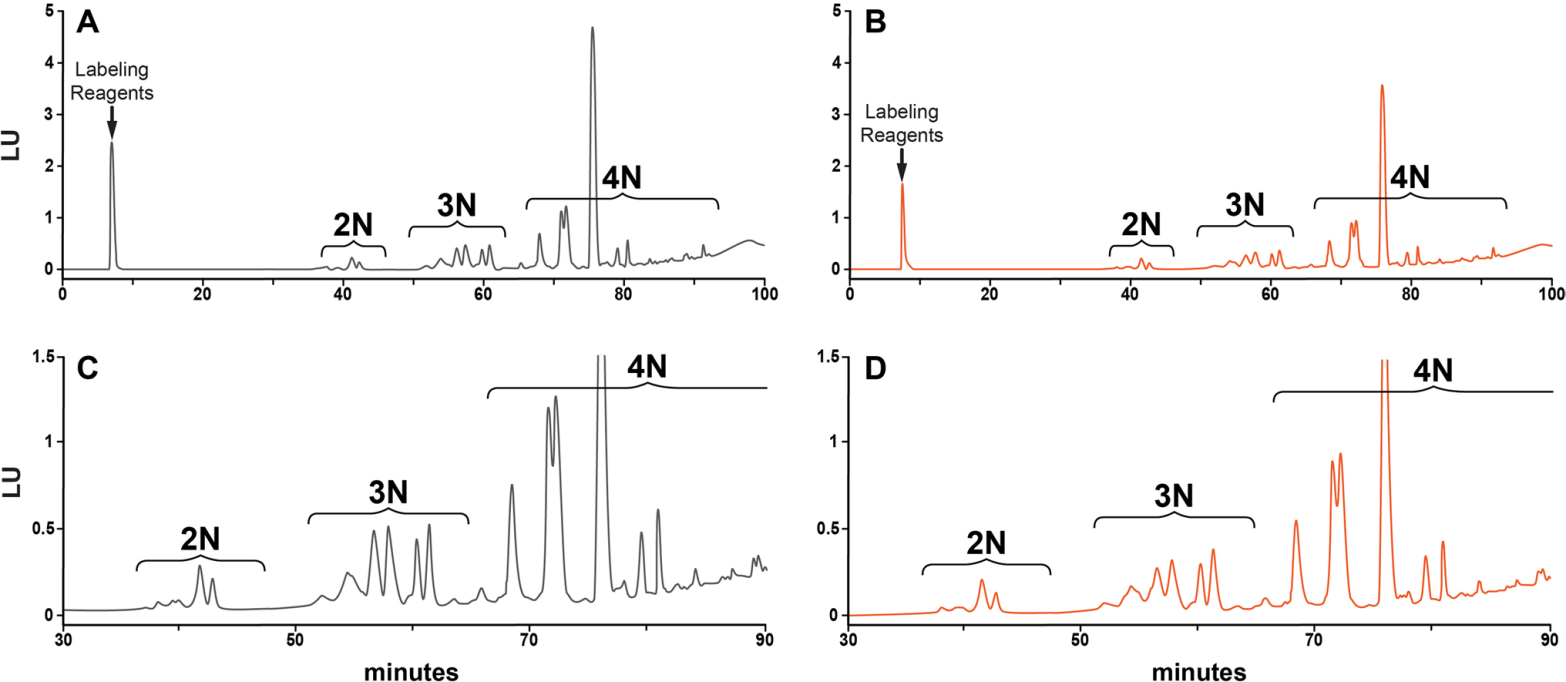
*N*-glycan analysis. 100 μg of rHuEPO-DS (grey) and USP rHuEPO-RS (orange) were treated with PNGase to remove the *N*-glycans. The free *N*-glycans were separated by HPLC. (A) rHuEPO-DS, (B) USP rHuEPO-RS buffer exchanged, (C) close-up view (3x) of rHuEPO-DS, (D) close-up view (3x) of USP rHuEPO-RS. Bi-sialylated (2 *N*), tri-sialylated (3 *N*), and tetra-sialylated (4 *N*) glycans are bracketed.

A significant amount of the rHuEPO’s mass is due to carbohydrates added through glycosylation. (17) *N*-linked glycans, composed predominantly of the tetra-antennary structure with and without repeating *N*-acetyllactosamine units, are covalently attached to Asparagine 24, 38, and 83 (Supplementary Fig. 1), and one *O*-linked glycan is attached to Serine 126. (18, 19) For this study the glycosylation of rHuEPO-DS and USP rHuEPO-RS was assessed by enzymatically removing the *N*-glycans, then labeling and separating them as described. The glycoprofile of both rHuEPO samples were evaluated by comparing peak areas and retention times of the eluting glycans (Fig. 4A through D). The integrated peak areas and the percent contribution of each group (2 *N*–4 *N*) to the total peak area are shown in Table IV. The *N*-glycans attached to rHuEPO are highly sialylated tetra-antennary complex types. They are grouped into bi-sialylated *N*-glycans (2 *N*), tri-sialylated *N*-glycans (3 *N*), and tetra-sialylated N-glycans (4 *N*). Mono-sialylated forms are also present, albeit in low quantities. All the major sialylated *N*-glycans (2 *N*–4 *N*) determinations were consistent between rHuEPO-DS and USP rHuEPO-RS.

**Table IV:** 2 *N,* 3 *N, and* 4 *N* glycans in rHuEPO-DS and rHuEPO-RS

	2 *N* (%)	3 *N* (%)	4 *N* (%)
**rHuEPO-DS**	5.36 ± 0.05	21.55 ± 0.07	73.07 ± 0.05
**USP rHuEPO-RS**	5.28 ± 0.22	21.53 ± 0.15	73.27 ± 0.42

The percentages for rHuEPO-DS are the averages of three separate *N*-glycan profiling runs. The percentages for USP rHuEPO-RS are the average of two separate runs performed by each of three separate participants in a multi-laboratory study. The standard deviations were calculated and are shown. Bi-sialylated (2 *N*), tri-sialylated (3 *N*), and tetra-sialylated (4 *N*) glycans

### Isoelectric Focusing

The host cell and bioreactor conditions used for production determine the quantity and type of post-translational modifications. For example, synthesizing rHuEPO in Chinese hamster ovary (CHO) cells results in five glycosylated isoforms labeled 10 through 14 to designate the number of sialic acids present. The glycans used by CHO cells are highly negatively charged, and they significantly influence the pH at which rHuEPO isoforms will have no net electrical charge. Any variation in growth conditions that perturbs the CHO cells can affect the pattern of rHuEPO glycosylation. Therefore isoelectric focusing (IEF), which separates the isoforms based on their charge differences, can be used to monitor this attribute. (20, 21) The resulting isoelectric profiles will differ between rHuEPOs produced under different conditions, revealing any variability in the manufacturing process. (22)

**Fig. 5:**
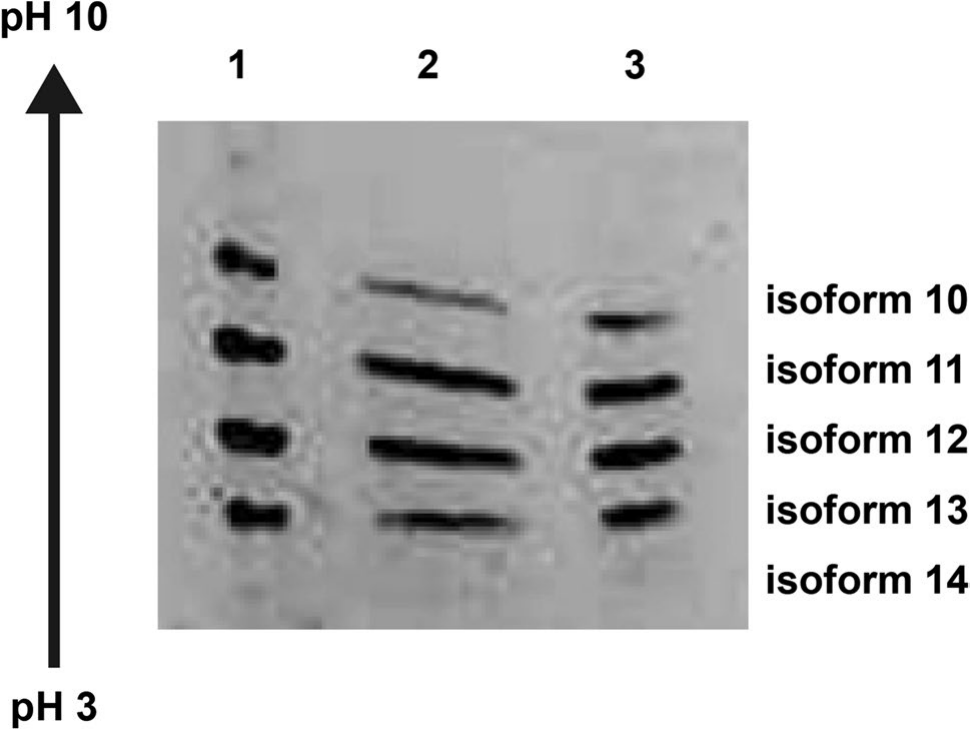
Isoelectric focusing. 20 μg of rHuEPO-DS and USP rHuEPO-RS were separated by IEF (pH 3 to 10). Each sample separates into 5 isoforms (isoforms 10–14) separated by the number of attached sialic acids. rHuEPO-DS (lane 1), USP rHuEPO-RS reformulated in water (lane 2), and USP rHuEPO-RS buffer exchanged (lane 3).

In this study, IEF was performed on samples of rHuEPO-DS and USP rHuEPO-RS in polyacrylamide slab gels. (23–25) The rHuEPO isoforms were detected with Coomassie stain (Fig. 5). rHuEPO-DS showed distinct compact bands, but USP rHuEPO-RS displayed elongated and wavy bands. An aliquot of lyophilized USP rHuEPO-RS (20 μg) was resuspended in water and buffer exchanged into citrate buffer to remove any excipients from lyophilization. IEF was performed on the buffer exchanged USP rHuEPO-RS. The resultant Coomassie stained bands were more compact and similar in appearance to the rHuEPO-DS. The gel was analyzed, and each isoform’s relative percentages were calculated as described (Table V).

**Table V:** Charge Variant Distribution of rHuEPO-DS and rHuEPO-RS

	Relative % of each isoform				
Isoform	10	11	12	13	14
rHuEPO-DS	16.04	28.74	31.26	22.75	1.21
USP rHuEPO-RS (buffer exchanged)	11.62	30.49	31.57	24.50	1.83

Isoforms 10–11 are designated by the number of sialic acids on each rHuEPO molecule

## Conclusion

Many manufacturers develop *in-house* standards for their products. However, developing reference standards (RS) to support analytical testing across multiple manufacturers is also essential to ensure the development of equivalently bioactive and safe drug products. A collaborative process between standards-setting bodies, manufacturers, and regulators is the best way to create confidence that any commercially available RS is publicly vetted and fit for its intended purpose. (26) A universally accepted RS also supports comparability testing in post-approval changes.

Recombinant human erythropoietin (rHuEPO) is prescribed to treat renal anemia associated with chronic renal failure and chemotherapy treatments. (27) The demand for rHuEPO has created a significant burden on current manufacturers and incentives for new biosimilar entrants. However, scaling up rHuEPO production or developing a new process is challenging because the product is manufactured in cell culture and has multiple post-translational modifications that must be monitored to achieve safety and efficacy.

In this study, we reformulated donated rHuEPO drug substance (rHuEPO-DS) for lyophilization to make a reference standard amenable to long-term storage at −20°C. This rHuEPO reference standard (USP rHuEPO-RS) was then characterized and compared to rHuEPO-DS using methods described previously. The analytical methods included peptide mapping to confirm identity, analysis of monodispersity and glycan profiling, and isoelectric focusing to monitor post-translational modifications.

During the initial peptide mapping, we observed that the newly formulated reference standard differed from the bulk drug substance. Buffer exchange of the resuspended lyophilized USP rHuEPO-RS demonstrated that a high concentration of excipients could interfere with peptide mapping experiments. It was further shown that excipients could interfere with other critical quality attribute tests, such as *N*-glycan analysis. Reducing the concentration of excipients in reconstituted lyophilized USP rHuEPO-RS by buffer exchange before testing brought the critical quality attributes (CQAs) for these analyses into alignment with the bulk drug substance results.

The USP rHuEPO-RS we describe here has been developed through a multilaboratory study. The extensive characterization of the material, as well as the methods and results presented in this manuscript, demonstrates good control over inter-laboratory variability, which is crucial for achieving consistent performance between different manufacturers. Additionally, the attributes of the RS are aligned with the CQAs of the commercially available rHuEPO drug substance. On this basis, we believe that USP rHuEPO-RS can be an important tool for developing methods used to validate the quality attributes of manufactured rHuEPO products. Validating is important because attributes can drift, even for biologics transferred between licensing partners. (28) For example, testing different commercially produced rHuEPO products has shown that biological activity can vary as much as 70% to 200% of the stated specifications. (29) In another study using IEF, a dozen rHuEPO products from manufacturers in Korea, Argentina, China, and India varied substantially in molecular weights. (30)

The USP rHuEPO-RS is also useful for demonstrating the system suitability of equipment used for analytical purposes. For example, many of the chromatographic methods used to analyze rHuEPO were initially developed to analyze highly purified rHuEPO monomeric proteins or to investigate EPO metabolic pathways, not for comparability studies. (31–33) We demonstrate here that the USP rHuEPO-RS combined with SEC-HPLC offers a robust method to measure total aggregates on a routine basis with high sensitivity for use in product quality control. (34)

In summary, USP rHuEPO-RS, along with EPO Chemical Reference Substance (CRS) developed by the European Pharmacopoeia, helps to underpin the global framework of quality of methods and equipment used to manufacture rHuEPO products. (35, 36) Each is validated against a separate yet overlapping set of physicochemical methods, thereby providing biosimilar manufacturers with validated standards that satisfy both US and European regulators. (36) Combining these standards with USP’s Erythropoietin Bioassay reference standard for measuring potency gives manufacturers tools for maintaining the quality of their EPO products, which can help reduce the barriers that block access to more EPO products and stimulate competition among manufacturers.

## Supplementary Information

Supplementary Figure 1. rHuEPO is an up–up–down–down four-helical bundle. The α-helices αA (residues 8–26), αB' (residues 47-52), αB (residues 55–83), αC (residues 90–112), αC' (residues 114-121), and αD (residues 138–161) are shown as orange cylinders. β-sheets β1 (residues 39-41) and β2 (residues 133-135) are shown as orange ribbons. The two disulfide bonds (Cys 7 to Cys 161 and Cys 29 to Cys 33) are denoted by dashed lines. The *N*-linked glycosylation sites (Asn 24, 38, and 83) are denoted by spheres or circles. (JPG 1977 kb)

## Declarations

### Disclaimer

Certain commercial equipment, instrument, or materials are identified in this article to adequately specify the experimental procedure. Such identification does not imply approval, endorsement, or certification by the United States Pharmacopeial Convention (USP) of a particular brand or product, nor does it imply that the equipment, instrument, or material is necessarily the best available for the purpose, or that any other brand or product was judged to be unsatisfactory or inadequate.
