# Supplementary figures and images for: A Reference Standard for Analytical Testing of Erythropoietin
Source: Pharm Res. 2022 Mar 15;39(3):553–62. doi: 10.1007/s11095-022-03213-1 (PMC8986685; doi:10.1007/s11095-022-03213-1)

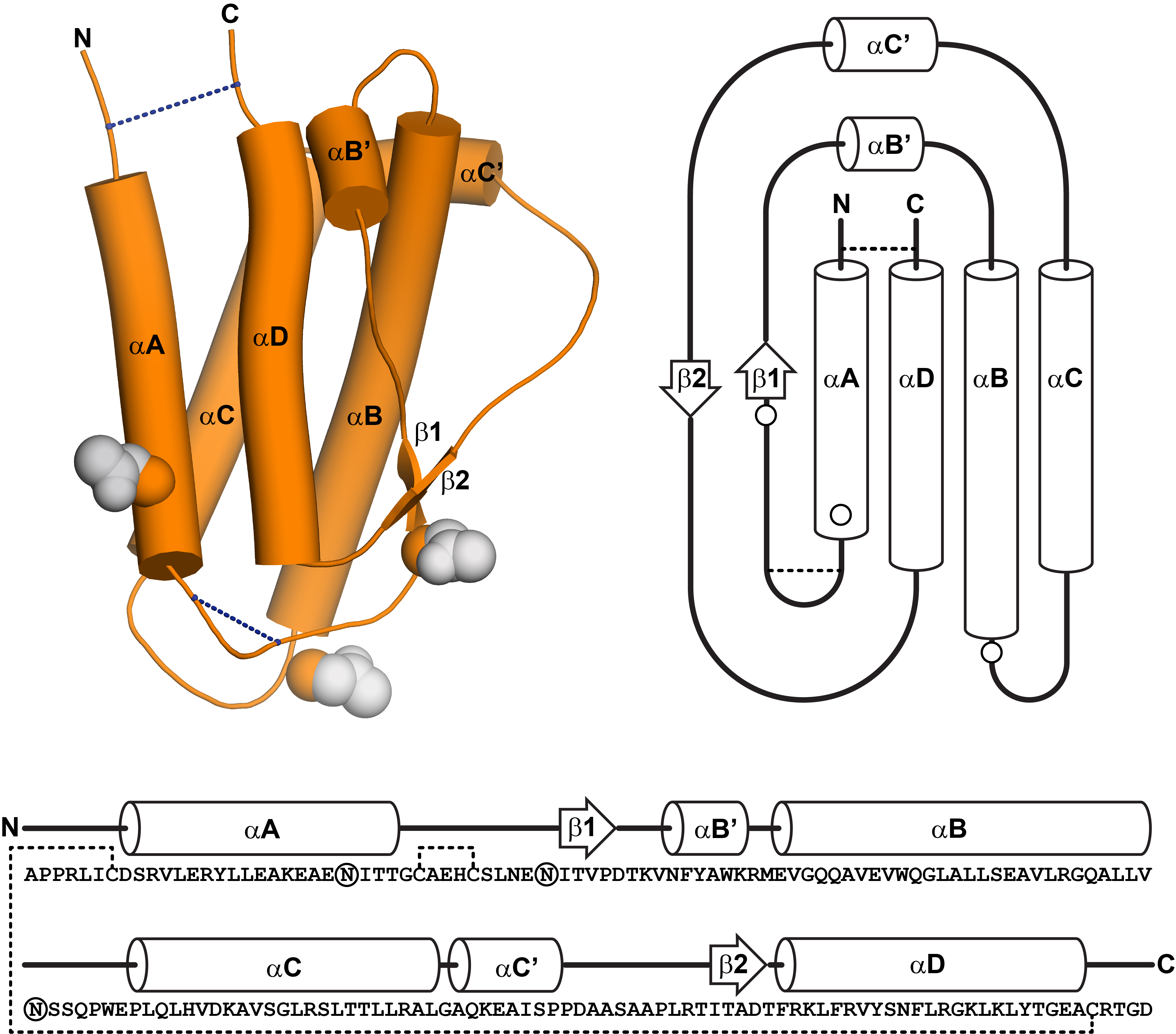

Supplement: Supplementary file 1 — rHuEPO is an up–up–down–down four-helical bundle. The α-helices αA (residues 8–26), αB' (residues 47-52), αB (residues 55–83), αC (residues 90–112), αC' (residues 114-121), and αD (residues 138–161) are shown as orange cylinders. β-sheets β1 (residues 39-41) and β2 (residues 133-135) are shown as orange ribbons. The two disulfide bonds (Cys 7 to Cys 161 and Cys 29 to Cys 33) are denoted by dashed lines. The N-linked glycosylation sites (Asn 24, 38, and 83) are denoted by spheres or circles. (JPG 1977 kb) [file 11095_2022_3213_MOESM1_ESM.jpg]
